# Hemodynamic Changes in the Masseter and Superior Orbicularis Oris Muscles before and after Exercise Load: A Comparison between Young Adult Women and Middle-Aged to Old Adult Women

**DOI:** 10.1155/2022/5340301

**Published:** 2022-08-27

**Authors:** Keiko Okamoto, Chihiro Tanikawa, Kenji Takada

**Affiliations:** ^1^Global Center for Medical Engineering and Informatics, Osaka University, Osaka, Japan; ^2^Department of Orthodontics and Dentofacial Orthopedics, Graduate School of Dentistry, Osaka University, Osaka, Japan

## Abstract

**Background:**

The vascularity index (VI) is useful for measuring the hemodynamics on ultrasound imaging. However, there are no reports concerning the application of the VI to facial muscles.

**Objective:**

The aim of this study was (1) to establish a method of measuring the hemodynamics in facial muscles in a constant way and (2) to evaluate the hemodynamic changes in the masseter and superior orbicularis oris muscles (SOOMs) before and after exercise load in two subject groups of females of different ages.

**Methods:**

(1) The VI in the SOOM was calculated, and the test-retest reliability was assessed in seven healthy adults. (2) The VIs in the left-side masseter and SOOM were calculated in 3 sessions: before exercise loading (*T*0), immediately after loading (*T*1), and 5 minutes after *T*1 (*T*2) for the young adult group (YAG, *n* = 20; age range, 20–35 years) and the middle-aged to old group (MOG, *n* = 20; age range, 50–70 years). Tasks were gum chewing for the masseter muscle and lip sealing for the SOOM. The differences in the mean peak flows between two sessions were examined.

**Results:**

(1) Significant differences were not noted for the repeatedly measured average volumes of blood flow with good test-retest agreement (intraclass correlation coefficient = 0.81). (2) In both muscles of the YAG, there were a significant increase in *T*1 compared with *T*0 and a significant decrease in *T*2 compared with *T*1 (all *p* < 0.05). In both muscles of the MOG, no significant differences were noted in either comparison.

**Conclusions:**

A method of measuring the hemodynamics in facial muscles was developed and showed good reliability. Changes in the blood flow after exercise load in these muscles may vary with age in women.

## 1. Introduction

In humans, the mouth is an important organ not only as the beginning of the digestive system (i.e., feeding, chewing, and swallowing) but also for verbal and nonverbal communication (i.e., speaking and smiling). For these purposes, the masticatory and perioral muscles work together as both digestive and communicative organs.

Hypofunction in the masticatory and perioral muscles has been suggested in several studies [1–4]. Hypofunction of these muscles was shown to be mainly caused by two factors: disuse atrophy and/or aging. Skeletal muscle atrophy, mainly represented by a decrease in the muscle mass, metabolic activity, and changes in the type of muscle fibers, occurs in response to a decrease in external loading, neural activation, and malnutrition [5]. Atrophy of the masticatory muscle has been reported following intracranial trigeminal schwannoma resection, pure trigeminal motor neuropathy, and a masticatory habit of unilateral chewing [1, 3, 4]. Furthermore, an age-related decline in the facial movement has been reported in older women [2]. The mechanisms underlying facial aging are considered to involve the external (skin) and intrinsic (muscles, nerves, subcutaneous fat) layers and their interactions based on hormonal and genetic regulation [6, 7]. In fact, the changes associated with advanced age in humans include significant changes in the skeletal muscle structure and function, such as sarcopenia [8, 9], a reduced oxidative capacity [9, 10], and fewer capillaries surrounding muscle fibers [8, 9, 11].

Given the above, it has been assumed that hypofunction in the masticatory and perioral muscles is due to a combination of disuse atrophy and aging. To date, however, few methods of measuring hypofunction in these muscles in a clinical setting have yet been established. Several studies have shown that mastication induces physiological changes in the perioral organs [12–14]. This suggests that hemodynamics can be an indicator for evaluating hypofunction in the masticatory and perioral muscles.

In particular, orthodontic patients with malocclusion have been shown to have an impaired masticatory efficiency, and indeed, a decreased smoothness in the trajectory of the jaw movement has been observed in these patients [15]. Experimentally induced malocclusion is known to impede the electromyography activity of the masseter [16] and smooth jaw movements [17, 18] during mastication, which is related to the metabolic activity of the masseter. Correcting malocclusion by orthodontic treatment was shown to significantly improve the smoothness of the trajectory of the jaw movement [19, 20]. Based on these findings, it is hypothesized that the presence of malocclusion reduces the metabolic activity, represented by the blood flow of the masticatory and perioral muscles, which may induce disuse atrophy of the oral muscles. Thus, it is important for orthodontists to understand the metabolic activity of the masticatory and perioral muscles, as orthodontic treatment may contribute to the recovery of the decreased strength of the masticatory muscle.

The vascularity index (VI) has been proposed as an index for measuring hemodynamics. The VI is known to be useful for measuring the blood circulation on ultrasound imaging and has been used to assess rheumatic arthritis [21], thyroid disease [22], gastric cancer [23], and ovarian cancer [24]. Several studies have used the VI to evaluate the intramuscular blood flow in appendicular skeletal muscles [25–27]. However, the VI has not yet been used to assess the masticatory and perioral muscles.

There is considerable evidence that actively metabolizing cells around arterioles release vasoactive substances that cause vasodilation. The mechanism that connects blood flow and metabolism involves changes in the partial pressure of oxygen. Two main routes for the metabolic regulation of the blood flow have been proposed: via hypoxia and tissue metabolites (e.g., adenosine, carbon dioxide, and lactic acid) and via ions (e.g., potassium ion and hydrogen ion). The relationship between capillary growth and metabolic activity has been studied extensively in skeletal muscles [28, 29]. We therefore hypothesized that the VI could be used as an indicator to assess the hemodynamics and metabolism in the masticatory and perioral muscles and that the hemodynamics of these muscles might be influenced by aging and/or atrophy.

This study was undertaken with two aims: to establish a method for measuring the hemodynamics in the masticatory and perioral muscles in a constant manner and to examine variations in the nature of the hemodynamic changes in the masseter and superior orbicularis oris muscles (SOOMs) before and after exercise load between young and middle-aged to old female individuals.

## 2. Materials and Methods

### 2.1. Testing the Validity of the Method

#### 2.1.1. Samples

Seven healthy adult female volunteers between 20 and 35 years old were included. We decided to target females in this study because there are more female orthodontic patients than male orthodontic patients. The inclusion criteria were as follows: no congenital facial deformities, including cleft lip and/or palate; no facial paralysis; no noticeable scars or skin diseases of the neck or dentofacial regions (or history thereof); no history of any psychiatric disorder; no subjectively or objectively discernible jaw hypofunction; and a body mass index ranging from 18.50 to 24.99.

A written informed consent form was distributed to and signed by all participants prior to their involvement in the study. This study was reviewed and approved by the ethics committee of the Global Center for Medical Engineering and Informatics, Osaka University (No. 1).

#### 2.1.2. Data Acquisition

**Figure 1:**
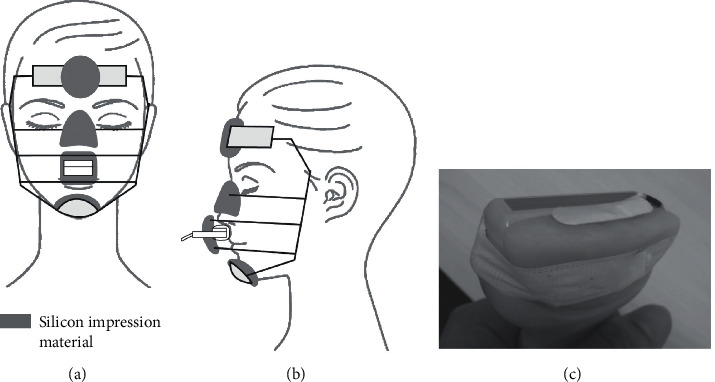
(a, b) A facemask was used for the measurement of the superior orbicularis oris muscle. Silicone impression material was placed onto a headrest, a chin cup, and an upper bow positioned above the root of the nose and fitted to the face in order to ensure accurate repositioning to the same position. (c) A pressure sensor was attached to a mounting base and fixed with adhesive tape.

The experiment was conducted in a quiet, air-conditioned room with a room temperature of 24–27°C and 46%–62% humidity. The blood flow of the SOOM was measured with an ultrasonic probe (PLT-1204BT; 14.0–7.2 MHz) supported by ultrasonic diagnostic equipment (Aplio 300; Canon Medical Systems Corporation, Tochigi, Japan). Participants were asked to lie down on their back on a reclining chair and instructed to maintain a resting condition. The probe was fixed to a face mask with silicone impression material (Genie; J. Morita Corp., Osaka, Japan) to secure the position and contact pressure of the probe with respect to the skin surface. Silicone impression material was placed onto a headrest, a chin cup, and an upper bow positioned above the root of the nose and fitted to the face to ensure accurate repositioning to the same position. To monitor the contact pressure of the probe to the skin surface, the contact pressure sensor of a measuring device (AMI3037-SB; AMI Techno Corporation, Tokyo, Japan) was attached to a mounting base made of polymerized acrylic resin, adjusted to the level of an acoustic lens surface, and fixed to the probe with adhesive tape (Figure 1). The probe and sensor were covered with the ultrasound gel pad (UACK-003A; Canon Medical Systems Corporation) to secure a clear image. They were positioned perpendicular to the upper lip by visual inspection and fixed to both the middle bow and lower bow.

**Figure 2:**
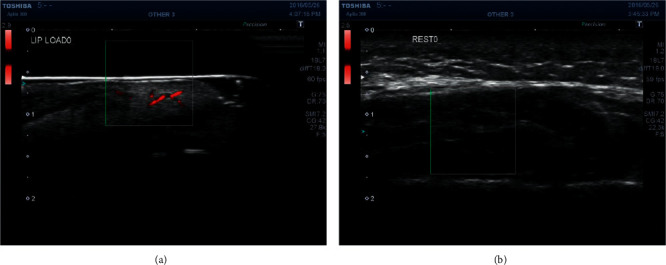
(a) Deeper half of the region of interest (ROI) was matched to the superior orbicularis oris muscle. (b) The ROI was included in the masseter.

Colored superb microvascular imaging (SMI) mode, a Doppler imaging technique, was used. The center of the region of interest (ROI) was located at almost the median value. The deeper half of the ROI was matched to the SOOM. The size of the ROI was 10 × 10 mm (Figure 2).

To confirm the repeatability, recordings were made four times (sessions *R*0, *R*1, *R*2, and *R*3). In Session *R*0, the blood flow was recorded after 15 minutes of rest. Each recording was conducted for 20 seconds, with a sampling rate of 30 frames/second. The probe was held in a fixing device to maintain a constant contact pressure during recordings. The probe and the fixing device were held over the face by the examiner during the first interval (between *R*0 and *R*1) but were removed from the face during the remaining intervals.

#### 2.1.3. Data Processing and Analyses

The stored data were transferred to a computer and analyzed using the MATLAB software program (The MathWorks, Inc., MA, USA). The VI, defined as the number of colored pixels due to the blood flow signal in the ROI/total number of pixels in the ROI × 100 (%), was calculated for each frame dataset. The mean VIs were determined for *R*0, *R*1, *R*2, and *R*3. The intraclass correlation coefficients (ICCs) were used to assess the test-retest reliability among these time points (1,1). These mean VIs were also compared statistically with a repeated-measures analysis of variance (ANOVA), as the robustness of a repeated-measures ANOVA in small samples has been confirmed even with a sample size as small as 3 [30]. The errors in the repeated measurements were calculated, with the average value set as the gold standard for each sample. The errors were expressed as a percentage with the following equation: (the standard deviation among the repeated measures)/(the average value among the repeated measures) × 00 (%).

All statistical analyses were performed using the SPSS Statistics software program, version 28 (IBM, Armonk, NY, USA).

## 3. Comparisons between the Two Age Groups

### 3.1. Samples

Twenty young female adults between 20 and 35 years (young adult group (YAG)) and 20 middle-aged to old female adults between 50 and 70 years (middle-aged to old group (MOG)) were enrolled. The inclusion criteria, informed consent forms, and institutional ethics committee approval were the same as mentioned above for this study.

### 3.2. Tasks

For the masseter muscle measurements, the task was gum chewing for two minutes on the left side. The duration of gum chewing was decided based on our previous research showing an increase in facial temperature after gum chewing for two minutes due to an increase in blood flow [14]. For the SOOM measurements, participants were asked to perform a lip sealing task using a lip training device (Japan Dental Supply Corp., Tokyo, Japan) for 1 minute. The strength of the spring was 400 g.

### 3.3. Data Acquisition

The environment of the experimental room was described in *1.2 Data acquisition*. Each participant was seated on a reclining chair and equipped with an ultrasound recording device.

Recordings were performed in the order of the masseter first, followed by the SOOM. The blood flow to the SOOM was measured using the same method as described in the preceding part of the current report. For the measurement of the superficial and deep parts of the masseter, the method for positioning the devices was modified as follows: (1) the bows of the fixing device were adjusted to secure the probe over the facial skin surface overlying the left-side masseter muscle, (2) the ultrasound gel pad was not used because the image of the masseter was able to be recorded clearly without the pad, and (3) the ROI was included in the muscle (Figure 2). The position of the masseter muscle was confirmed by palpation and visual inspection on the ultrasound monitor [31].

The blood flow was recorded in three sessions (*T*0, *T*1, and *T*2) in a consecutive manner. Session *T*0 was recorded after taking a five-minute rest. The probe was held in the fixing device to maintain a constant level of contact pressure during recording. After *T*0, the probe and fixing device were removed from the face. Each participant was then asked to perform the tasks. Immediately after the task, the probe and fixing device were repositioned on the face, and *T*1 was recorded. Five minutes later, *T*2 was recorded. The probe and fixing device were held over the face by the examiner during the interval between *T*1 and *T*2. Each session was recorded for 20 seconds, with a sampling rate of 30 frames/second.

### 3.4. Data Processing and Analyses

The stored data were transferred to a computer and analyzed. The VIs were calculated as above. The peak flow was defined as the highest VI value in periodically changing VIs. Mean peak flows were compared statistically between *T*0 and *T*1 and between *T*1 and *T*2 using a paired *t*-test (*p* ≤ 0.05). Subsequently, Benjamini and Hochberg's multiple-comparison test was performed.

## 4. Results

### 4.1. Measurement Repeatability

**Figure 3:**
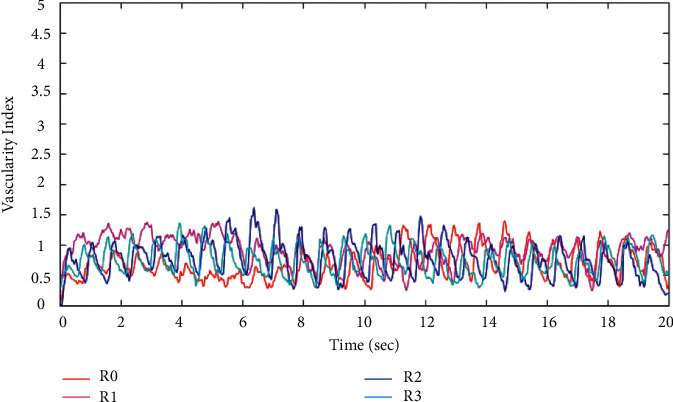
Vascularity index (VI) calculated from images of one participant.

In the first experiment, the test-retest reliability determined by the ICC was 0.812 (Table 1), suggesting good agreement [32]. Significant differences were not detected for the repeatedly measured average volumes of blood flow (*p*=0.8). The variation in the repeated measures was 0.21% ± 0.22% (Figure 3).

### 4.2. Comparisons between the Two Age Groups

**Figure 4:**
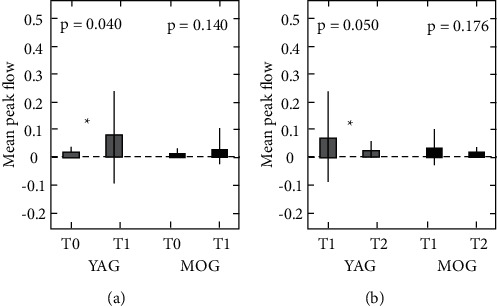
Mean peak flow measured in the masseter. Numeric values are shown as the means ± SDs. ^*∗*^*p* < 0.05 comparing (a) *T*0 and *T*1 and (b) *T*1 and *T*2. *T*0, before the task; *T*1, immediately after the task; *T*2, five minutes after *T*1; YAG, young adult group; MOG, middle-aged to old group.

For the masseter muscle, the mean peak flows were 0.008 ± 0.009 (T0), 0.040 ± 0.065 (*T*1), and 0.010 ± 0.010 (*T*2) in the YAG and 0.015 ± 0.015 (*T*0), 0.072 ± 0.016 (*T*1), and 0.025 ± 0.032 (*T*2) in the MOG. In the YAG, there were a significant increase in *T*1 compared with *T*0 and a significant decrease in *T*2 compared with T1 (*p* ≤ 0.05). The MOG showed the same tendency as the YAG. However, no significant differences were noted between *T*0 and *T*1 or *T*1 and *T*2 (Figure 4).

**Figure 5:**
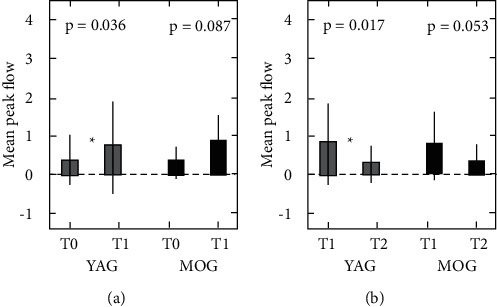
Mean peak flow measured in the superior orbicularis oris muscle. Numeric values are shown as the means ± SDs. ^*∗*^*p* < 0.05 comparing (a) *T*0 and *T*1 and (b) *T*1 and *T*2. *T*0, before the task; *T*1, immediately after the task; *T*2, five minutes after *T*1; YAG, young adult group; MOG, middle-aged to old group.

For the SOOM, the mean peak flows were 0.44 ± 0.55 (*T*0), 0.86 ± 0.82 (*T*1), and 0.44 ± 0.44 (*T*2) in the YAG and 0.33 ± 0.59 (*T*0), 0.76 ± 1.10 (*T*1), and 0.29 ± 0.44 (*T*2) for the MOG. In the YAG, there were a significant increase in *T*1 compared with *T*0 and a significant decrease in *T*2 compared with *T*1 (*p* < 0.05). The MOG showed the same tendency as the YAG. However, no significant differences were noted between *T*0 and *T*1 or *T*1 and *T*2 (Figure 5).

## 5. Discussion

In this study, we successfully established a method of measuring the hemodynamics in the human masseter and SOOM before and after exercise loading using the VI in a constant manner. The second experiment showed that the hemodynamic changes after exercise were significantly different between young and middle-aged to old adults. The details are described as follows.

### 5.1. Method of Holding the Ultrasound Probe

In general, an ICC ≥0.6 is necessary and ICC ≥0.8 is considered preferable when assessing the reliability of continuous data [32]; notably, our first experiment showed an ICC of 0.81. Furthermore, no significant differences were noted for the repeatedly measured average volumes of the blood flow (*p*=0.8). The error in the repeated measures was 0.2%, suggesting high measurement repeatability. Generally, in ultrasound imaging, images can easily change due to the position and incidence angle of the probe. Whenever the tester changes the hand force magnitude used to press the probe over the skin, the obtained images can waver easily. Therefore, when measuring the intramuscular blood flow before and after exercise loading, the position, incidence angle, and contact pressure must be kept constant [25].

Previous reports have described techniques used to maintain a constant position of the probe, such as a marker [33] and a fixing device [25, 34]. In this study, it was necessary to remove the probe temporarily from the face between the experimental sessions. Therefore, we designed and employed a fixing appliance with a contact pressure-measuring device incorporated into it that could be easily restored to the initial fixing position. The assessment of the intramuscular blood flow before and after orthodontic treatment or muscle training is thus feasible using such a fixation device.

### 5.2. Comparisons between Young and Middle-Aged to Old Adults

In this study, both the YAG and MOG showed the same trends in hemodynamic changes in the masseter and SOOM before and after exercise loading. However, significant differences were detected only in the YAG. When measuring the masseter in the YAG, there were a significant increase in T1 (after load) compared with *T*0 (rest) and a significant decrease in *T*2 (5 minutes *T*1) compared with *T*1. In contrast, no significant differences were noted between *T*0 and *T*1 or between *T*1 and *T*2 for the MOG. Similar results were shown when measuring the SOOM. These results suggested that young adults show more marked hemodynamic changes than middle-aged to old adults in both the masseter and SOOM.

When considering hemodynamics in the masseter and SOOM, it is important to take into account the differences in the muscle fiber type composition. A human skeletal muscle fiber is classified roughly into two types: slow-twitch type I and fast-twitch type II. Type II is further subdivided into types IIa, IId/*x*, and IIb. The masseter contains more type I fibers than type II fibers [35–40], while the orbicularis oris muscle contains more type II fibers than type I fibers [41]. Several studies have shown that the blood flow and oxygen consumption are greater in slow muscle (type I) than in fast muscle (type II) [42], while type IIa muscle fibers contain abundant glycogen and mitochondria [43, 44] to ensure adequate ATP generation and compensate for the accelerated rate of ATP hydrolysis in these fast-twitch fibers. This is related to the fact that type I fibers generally have higher capillary densities than type II fibers [42, 45, 46].

There have been several reports that type I fibers are predominantly affected by disuse [47], whereas type II fibers are predominantly affected by aging [48]. A previous report showed that aging can reduce the muscle fiber cross-sectional area (FCSA) of type II fibers in humans [8, 9]. In addition, in a report investigating fiber type-specific muscle capillarization in humans [9], aged men showed a lower capillary-to-fiber ratio (C/F) in IIa and IIb fibers than young men, although no such reduction was noted in type I fibers. Age-associated reductions in muscle capillarization may be present predominantly surrounding type II muscle fibers in humans. The intrinsic aging of muscles is reported to be hormonally and genetically regulated and responsible for soft tissue remodeling [49].

The changes in the FCSA observed in response to disuse have been well studied in rodent models. In certain rodent muscles, the reduction in the FCSA can first be seen in slow type I fibers, followed by fast type IIa fibers and then fast type IIx and IIb fibers [50, 51]. In humans, differential atrophy across muscle fiber types has also been observed in response to disuse. For example, after prolonged disuse (e.g., roughly 180 days in cases of spaceflight), the loss of fiber size and force was reported in the soleus and gastrocnemius muscles, with the order of the effect being soleus type I > soleus type II > gastrocnemius type I > gastrocnemius type II [52]. A similar response was observed in the vastus lateralis muscle after 35 days of bed rest, where the loss of the FCSA was greater in type I fibers than in type II fibers [53].

These results suggest that the masseter (more type I) tends to suffer from disuse, while the orbicularis oris muscle (more type II) tends to be affected by aging. A summary of the previously published evidence is shown in Table 2. In this study, the YAG showed more apparent hemodynamic changes in both muscles than the MOG. Although it is difficult to distinguish disuse atrophy from aging by observing the VI, the present result indicates that changes in the blood flow in these muscles before and after exercise loading are useful indicators for examining the extent of metabolic decrease and thus for estimating any hypofunction in the masticatory or perioral muscles related to aging and atrophy.

### 5.3. Clinical Significance

Because chewing is the major function of the masticatory muscles, normal chewing activity seems to prevent muscle atrophy. In fact, a previous study showed that the facial temperature increased significantly during gum chewing by facilitating blood circulation and metabolic activity of the buccal and labial muscles [14], suggesting that chewing activity temporarily increases the metabolism in the masticatory muscles.

Hemodynamics are an indicator of the metabolic activity of skeletal muscles. Clinically, it is interesting to show the relationship between the hemodynamics of the facial muscles, aging, and interventions, such as orthodontic treatment or muscle training. Thus far, orthodontic treatment has been proven to improve the oral function, including the masticatory efficacy, and aesthetics. However, there is no evidence supporting the influence of orthodontic treatment on the metabolic activity of perioral muscles. The metabolic activity of facial muscle seems to increase after improving malocclusion. Interestingly, in this study, the YAG showed a greater standard deviation than the MOG at T1 (after workload) in the masseter, indicating that the YAG showed a greater variation in the hemodynamic response to the gum chewing tasks than the MOG. This might be related to individual malocclusion characteristics and the development of the masseter muscles. Future studies should focus on the hemodynamics before and after orthodontic treatment or muscle training.

### 5.4. Limitations

Several limitations associated with this study warrant mention. First, in the retest study, the number of participants was few, and retest in the masseter was not evaluated. Further studies will be necessary to establish more appropriate and accurate methods. Second, in the age comparison study, we did not measure the SOOM after the chewing task or the masseter after the lip sealing task. The results of these measurements would likely be interesting, so further studies will be needed. Finally, no experiments were conducted on male subjects. Further research is therefore needed to consider any possible differences due to sex.

## 6. Conclusion

The method proposed in this study showed good test-retest reliability when measuring the blood flow of the masseter and SOOM. It was suggested that changes in the blood flow in these muscles before and after exercise loading vary with age. Regarding the masticatory activity, the presence of malocclusion and improvements obtained by orthodontic treatment may affect the metabolic activity of the masticatory and perioral muscles. As a result, future studies should focus on the hemodynamics before and after orthodontic treatment or muscle training.

## Figures and Tables

**Table 1:** Test-retest reliability.

	ICC	95% CI	*p* value
Mean VIs	0.812	0.551–0.959	0.000

VI, vascularity index; ICC, intraclass correlation coefficient; CI, confidence interval.

**Table 2:** Summary of previously published research.

	Masseter	Orbicularis oris muscle
Main fibers	Type I (slow)	Type II (fast)
Consumption of oxygen	Greater	Smaller
Related to	Atrophy	Aging

## Data Availability

The data used to support the findings of this study are available from the corresponding author upon request.
